# Supplementary material for: Family-centered music therapy—Empowering premature infants and their primary caregivers through music: Results of a pilot study
Source: PLoS One. 2021 May 14;16(5):e0250071. doi: 10.1371/journal.pone.0250071 (PMC8121291; doi:10.1371/journal.pone.0250071)
Supplement: S1 Table — a. Main effects of within factor time on maternal stress factors. b. Main effects of between factor group on maternal stress factors. c. Interaction effects of within factor time and between factor group on maternal stress factors. (DOCX) [file pone.0250071.s001.docx]

**S1a Table. Main effects of within factor time on maternal stress factors.**

| Variable | F | p | η^2^_p_ |
| --- | --- | --- | --- |
| Stress | 20.33 | <.001 | 0.34 |
| Resources | 5.10 | .029 | 0.11 |
| State Anxiety | 11.70 | .001 | 0.21 |
| Trait Anxiety | 9.63 | .003 | 0.18 |
| Depression | 29.16 | <.001 | 0.39 |
| Skills | 23.66 | <.001 | 0.37 |

Note: df_1_=1, df_2_=45

**S1b Table. Main effects of between factor group on maternal stress factors.**

| Variable | F | p | η^2^_p_ |
| --- | --- | --- | --- |
| Stress | 0.33 | .856 | <0.01 |
| Resources | 1.70 | .199 | 0.04 |
| State Anxiety | 0.58 | .452 | 0.01 |
| Trait Anxiety | 0.11 | .745 | 0.02 |
| Depression | 2.52 | .120 | 0.53 |
| Skills | 0.81 | .777 | 0.02 |

Note: df_1_=1, df_2_=45

**S1c Table. Interaction effects of within factor time and between factor group on maternal stress factors.**

| Variable | F | p | η^2^_p_ |
| --- | --- | --- | --- |
| Stress | 0.24 | .624 | 0.01 |
| Resources | 0.39 | .537 | 0.01 |
| State Anxiety | 0.58 | .452 | 0.01 |
| Trait Anxiety | 0.05 | .825 | <0.01 |
| Depression | 0.60 | .443 | 0.01 |
| Skills | 0.00 | .995 | <0.01 |

Note: df_1_=1, df_2_=45
