# Supplementary material for: Family-centered music therapy—Empowering premature infants and their primary caregivers through music: Results of a pilot study
Source: PLoS One. 2021 May 14;16(5):e0250071. doi: 10.1371/journal.pone.0250071 (PMC8121291; doi:10.1371/journal.pone.0250071)
Supplement: S2 Table — a. Main effects of within factor time on paternal stress factors. b. Main effects of between factor group on paternal stress factors. c. Interaction effects of within factor time and between factor group on paternal stress factors. (DOCX) [file pone.0250071.s002.docx]

**S2a Table. Main effects of within factor time on paternal stress factors.**

| Variable | F | p | η^2^_p_ |
| --- | --- | --- | --- |
| Stress | 25.10 | <.001 | 0.47 |
| Resources | <0.01 | .965 | <0.01 |
| State Anxiety | 4.89 | .034 | 0.12 |
| Trait Anxiety | 2.85 | .101 | 0.80 |
| Skills | 0.84 | .0368 | 0.03 |

Note: df_1_=1, df_2_=45

**S2b Table. Main effects of between factor group on paternal stress factors.**

| Variable | F | p | η^2^_p_ |
| --- | --- | --- | --- |
| Stress | 0.14 | .716 | 0.01 |
| Resources | 0.15 | .903 | <0.01 |
| State Anxiety | 0.01 | .945 | <0.01 |
| Trait Anxiety | 0.46 | .501 | 0.01 |
| Skills | <0.01 | .997 | <0.01 |

Note: df_1_=1, df_2_=45

**S2c Table. Interaction effects of within factor time and between factor group on paternal stress factors.**

| Variable | F | p | η^2^_p_ |
| --- | --- | --- | --- |
| Stress | <0.01 | .952 | <0.01 |
| Resources | 3.21 | .084 | 0.10 |
| State Anxiety | 0.14 | .715 | 0.01 |
| Trait Anxiety | 0.46 | .501 | 0.01 |
| Skills | 0.00 | .997 | 0.00 |

Note: df_1_=1, df_2_=45
