# Supplementary material for: Family-centered music therapy—Empowering premature infants and their primary caregivers through music: Results of a pilot study
Source: PLoS One. 2021 May 14;16(5):e0250071. doi: 10.1371/journal.pone.0250071 (PMC8121291; doi:10.1371/journal.pone.0250071)
Supplement: S3 Table — a. Minimal dataset physiological development infants. b. Minimal dataset maternal stress factors. c. Minimal dataset paternal stress factors. (PDF) [file pone.0250071.s003.pdf]

S3a Table. Minimal Dataset Physiological Development Infants

No.	random	sex	GA	weight_birth	length_birth	HC_birth	age mother	length of stay	GA_discharge	weight_discharge	length_discharge	HC_discharge	caffein	tube-feed	MT Session
21	2	1	181	580	31,5	21,0	31	105	285	2620	45,0	32,5	86	101	0
24	2	2	164	530	30,0	20,5	19	112	289	2350	43,0	30,0	99	101	0
22	2	2	175	710	32,0	22,3	31	104	278	2655	46,0	34,0	95	98	0
13	2	2	162	340	27,0	19,6	37	122	283	2870	46,0	32,0	84	113	0
47	2	2	209	1650	44,0	30,0	38	36	244	2220	45,0	32,0	18	32	0
51	1	1	206	1130	38,5	26,3	33	45	250	2340	43,0	31,0	32	41	6
43	1	2	210	960	37,0	26,0	31	89	294	2255	45,5	31,5	80	86	16
25	2	2	206	980	36,0	27,0	39	88	263	2050	43,0	32,5	42	48	0
57	2	2	185	810	34,5	24,0	26	89	273	2500	44,0	33,5	78	84	0
55	1	1	190	1110	38,0	25,5	40	62	251	2400	45,0	32,5	39	50	9
42	2	1	187	720	33,0	23,3	38	73	259	2200	41,0	31,0	66	64	0
14	2	1	183	730	34,0	23,3	39	82	264	2400	43,0	31,6	62	74	0
53	2	1	188	900	30,5	24,3	20	81	268	2720	43,0	31,5	68	72	0
61	2	1	174	450	28,0	20,8	47	98	271	2250	43,0	30,5	76	87	0
16	1	1	201	1080	37,0	26,5	31	58	258	2190	45,0	32,0	42	49	8
44	1	1	210	1580	42,0	29,3	31	61	270	2760	49,0	34,5	31	51	9
12	1	2	195	990	37,0	25,0	24	64	258	2890	48,0	33,5	57	60	12
32	2	2	194	1190	39,0	27,0	32	93	286	3380	51,5	35,0	75	85	0
58	1	2	192	1010	37,0	26,2	37	72	263	2570	46,0	32,5	65	66	12
45	1	1	199	720	33,5	23,3	35	86	284	2415	42,5	31,6	71	83	17
18	1	1	186	640	34,0	22,0	31	96	281	2390	45,5	32,0	77	81	16
52	2	2	198	1360	37,0	26,0	42	48	245	2615	46,0	30,8	32	40	0
20	1	2	196	1450	41,0	26,5	37	60	255	2710	47,0	31,7	39	49	9
34	2	2	204	570	30,0	23,3	31	72	275	1980	40,5	32,5	41	65	0
64	1	2	194	950	36,0	26,0	26	82	274	2740	47,0	33,7	70	76	14

11	1	2	195	1160	36,0	22,5	30	68	262	2630	43,5	33,0	48	60	13
49	1	1	204	870	36,5	25,0	44	55	253	2640	43,0	32,5	28	50	9
23	1	2	199	1740	44,0	29,8	41	47	245	2975	49,0	34,0	19	41	6
48	1	2	210	1770	43,0	29,9	28	36	247	2610	48,5	33,6	29	33	7
19	1	1	196	820	32,0	24,0	40	76	271	2235	43,0	32,1	65	69	12
27	1	1	192	870	32,0	23,0	27	79	270	2395	45,0	31,5	71	73	14
10	1	2	195	1360	38,0	27,4	30	64	258	2910	46,0	33,5	48	55	13
39	2	2	195	1160	38,0	27,5	19	67	261	2680	46,0	34,0	33	62	0
41	2	1	183	520	30,0	22,7	37	91	273	2575	45,0	32,5	82	85	0
60	2	2	202	1480	42,0	28,5	38	59	260	2690	48,0	35,0	37	50	0
35	2	2	209	960	38,0	25,5	34	66	274	2100	42,0	32,5	43	49	0
36	1	1	198	1050	37,5	26,0	28	69	266	2375	43,0	33,2	46	63	10
59	2	1	202	1110	38,0	26,0	38	59	260	2315	45,0	33,0	37	49	0
54	2	2	174	820	34,0	23,5	36	104	277	3170	46,0	33,5	97	91	0
63	2	2	182	860	34,0	23,5	36	78	259	2435	47,0	32,0	61	68	0
38	1	1	188	950	36,0	24,0	29	77	264	2495	46,0	34,0	68	69	16
30	1	2	173	395	27,0	19,5	36	135	301	2580	46,0	33,5	88	132	11
46	2	2	210	920	33,0	24,7	38	57	266	2150	42,0	31,2	43	52	0
33	1	2	198	1490	40,0	27,0	40	53	250	2800	46,0	32,5	31	39	6
15	2	2	163	490	29,0	20,2	41	149	308	3790	50,0	35,5	94	135	0
29	1	2	194	990	37,5	25,0	35	62	255	2480	43,0	32,0	46	58	9
31	2	2	194	1200	36,0	26,2	32	89	282	3525	50,0	35,0	60	65	0
56	1	1	190	645	31,5	23,8	40	103	287	2670	44,0	33,5	93	99	14
17	2	2	176	750	31,5	22,5	43	123	294	3130	46,0	34,0	95	116	0
62	2	1	174	540	29,0	21,0	47	98	271	2300	43,0	31,5	75	88	0

Note: random: 1 = Music therapy group; 2 = Control group; sex: 1 = female; 2 = male; GA = gestational age; HC = head circumference; MT = Music therapy

S3b Table. Minimal Dataset Maternal Stress Factors

No.	random	sex	T1 Stress	T1 Resources	T1 StateAnxiety	T1 TraitAnxiety	T1 Depression	T1 Skills	T2 Stress	T2 Resources	T2 StateAnxiety	T2 TraitAnxiety	T2 Depression	T2 Skills
21	2	1	22,00	17,00	37,00	31,00	16,00	13,00	17,00	19,00	37,00	33,00	9,00	12,00
50	2	2	17,00	18,00	43,00	39,00	13,00	18,00	17,00	16,00	47,00	50,00	18,00	22,00
24	2	2	16,00	16,00	47,00	48,89	11,00	18,00	23,00	9,00	35,00	29,00	13,00	15,00
22	2	2	19,00	16,00	44,00	39,00	6,00	26,00	17,00	17,00	39,00	37,00	3,00	20,00
47	2	2	17,00	20,00	33,00	27,00	4,00	8,00	18,00	21,00	37,00	22,00	2,00	8,00
51	1	1	16,00	20,00	45,00	39,00	18,00	18,00	18,00	20,00	36,00	37,00	14,00	11,00
43	1	2	18,00	20,00	46,50	41,50	14,00	17,00	20,00	19,00	43,50	42,00	14,00	14,00
25	2	2	21,00	19,00	29,00	30,00	1,00	16,00	22,00	18,00	32,00	28,00	3,00	14,00
57	2	2	20,00	19,00	44,00	34,00	7,00	15,00	20,00	21,00	38,00	34,00	1,00	13,00
55	1	1	17,00	18,00	49,00	39,00	15,00	17,00	15,00	19,00	54,00	51,00	8,00	18,00
42	2	1	23,87	20,00	57,00	62,00	17,00	28,00	22,80	21,00	64,00	65,00	13,00	29,00
53	2	1	20,00	21,00	44,00	44,00	16,00	19,00	21,00	19,00	45,00	37,00	13,00	17,00
61	2	1	24,00	11,00	59,00	57,00	12,00	17,00	23,00	14,00	54,00	53,00	10,00	19,00
16	1	1	20,00	20,00	48,00	40,00	7,00	13,00	18,00	21,00	39,00	32,00	7,00	13,00
44	1	1	20,00	20,00	46,50	41,50	14,00	17,00	23,00	19,00	46,00	39,50	13,00	15,00
12	1	2	24,00	19,00	51,00	44,00	13,00	21,75	21,00	19,00	53,00	41,00	13,00	14,00
32	2	2	23,00	16,00	60,00	61,00	16,00	30,00	21,00	17,00	57,00	58,00	9,00	17,00
58	1	2	21,00	21,00	39,00	31,00	6,00	12,00	18,00	19,00	40,00	35,00	6,00	14,00
45	1	1	21,00	21,00	40,00	39,00	6,00	18,00	19,00	21,00	43,00	41,00	9,00	15,00
18	1	1	19,00	16,00	70,00	58,00	23,00	19,00	20,00	21,00	65,00	55,00	20,00	15,00
52	2	2	24,00	21,00	48,00	27,00	12,00	21,42	19,00	21,00	38,00	28,00	2,00	13,00
20	1	2	17,00	18,00	41,00	38,00	14,00	22,60	15,00	20,00	31,00	41,00	14,00	13,00
34	2	2	20,00	17,00	43,00	45,00	7,00	25,00	20,00	17,00	45,00	39,00	5,00	17,00
64	1	2	21,00	20,00	45,00	41,00	12,00	19,41	16,00	19,00	37,00	34,00	5,00	15,17
11	1	2	22,00	18,00	47,00	36,00	7,00	13,00	19,00	19,00	35,00	29,00	3,00	13,00
49	1	1	23,00	18,00	53,00	51,59	15,00	21,00	19,80	16,00	57,00	51,00	17,00	18,00

48	1	2	14,00	21,00	38,00	29,00	9,00	23,00	14,00	22,00	39,00	32,00	8,00	16,00
19	1	1	22,00	19,00	49,00	42,00	13,00	18,00	19,00	21,00	44,00	36,00	4,00	13,00
27	1	1	26,00	18,00	53,00	55,00	25,00	16,00	24,00	21,00	50,00	45,00	11,00	15,00
10	1	2	22,00	18,00	47,00	36,00	7,00	13,00	19,00	20,00	36,00	29,00	1,00	13,00
39	2	2	20,60	19,59	47,00	41,00	11,00	14,00	20,00	20,00	45,00	37,00	8,00	15,00
41	2	1	18,00	20,00	38,00	35,00	6,00	16,00	19,00	21,00	32,00	39,00	11,00	11,00
60	2	2	17,00	21,00	40,00	34,00	6,00	8,00	18,00	21,00	37,00	26,00	4,00	8,00
35	2	2	19,00	17,00	38,00	40,00	12,00	18,00	17,00	19,00	48,00	45,00	13,00	13,00
59	2	1	20,00	21,00	38,00	33,00	6,00	8,00	18,00	21,00	37,00	26,00	4,00	8,00
54	2	2	27,00	20,00	50,00	39,00	12,00	14,00	19,00	21,00	38,00	36,00	10,00	9,00
63	2	2	24,00	16,00	55,00	56,00	18,00	16,00	20,00	18,00	56,00	49,00	15,00	12,00
38	1	1	27,00	20,00	54,00	51,00	17,00	21,00	24,00	16,00	49,00	39,00	7,00	17,00
30	1	2	23,00	21,00	44,00	41,52	15,00	12,00	22,80	19,00	47,00	40,00	14,00	16,00
46	2	2	19,00	20,00	37,00	30,00	9,00	14,00	19,00	21,00	33,00	30,00	5,00	9,00
37	1	2	26,00	19,00	50,00	47,00	9,00	17,00	22,00	20,00	42,00	39,00	5,00	11,00
33	1	2	17,00	17,00	48,00	53,00	18,00	26,00	17,00	20,00	34,00	33,00	10,02	13,00
15	2	2	25,78	14,00	62,00	59,00	16,00	23,00	24,00	20,00	46,00	42,00	4,00	23,00
29	1	2	23,00	20,00	47,00	48,00	16,00	17,00	18,00	21,00	37,00	41,00	10,00	9,00
31	2	2	23,00	16,00	60,00	61,00	16,00	30,00	21,00	17,00	57,00	58,00	9,00	17,00
56	1	1	21,00	18,00	49,00	38,89	15,00	19,00	15,00	19,00	54,00	51,00	8,00	18,00
62	2	1	25,00	10,00	56,00	56,00	12,00	16,00	21,00	14,00	54,00	53,00	10,00	19,00

Note: random: 1 = Music therapy group; 2 = Control group; sex: 1 = female; 2 = male; * = missing data

S3c Table. Minimal Dataset Paternal Stress Factors

No.	random	sex	T1_Stress	T1_Resources	T1_StateAnxiety	T1_TraitAnxiety	T1_Skills	T2_Stress	T2_Resources	T2_StateAnxiety	T2_TraitAnxiety	T2_Skills
40	2	1	23,00	20,00	99*	99*	99*	22,00	21,00	99*	99*	99*
21	2	1	21,00	18,00	54,00	46,72	14,00	18,00	19,39*	44,53	40,00	15,00
24	2	2	23,00	17,00	38,00	29,00	33,00	20,00	9,00	41,51	26,00	8,00
22	2	2	18,00	17,00	40,00	33,00	16,00	17,00	18,39	23,53	22,00	12,00
51	1	1	16,00	19,00	31,00	27,00	13,00	14,00	19,39	28,00	22,00	11,00
43	1	2	99*	99*	41,00	34,00	21,65	99*	99*	47,00	35,00	10,00
25	2	2	24,00	17,00	52,00	46,00	21,00	21,00	18,00	35,00	45,00	19,00
57	2	2	20,00	21,00	46,00	36,00	13,00	21,00	23,00	46,00	39,00	14,00
55	1	1	21,00	19,00	53,00	53,00	17,00	21,00	18,00	51,00	54,00	20,00
42	2	1	21,00	19,00	48,00	46,00	15,00	21,00	18,00	41,00	40,49	11,00
53	2	1	20,00	17,00	38,00	40,72	23,00	17,00	16,39	43,53	46,00	28,00
16	1	1	18,00	20,00	43,00	37,00	18,00	16,00	20,39	29,00	27,00	15,00
44	1	1	99*	99*	41,00	35,00	21,65	99*	99*	44,00	39,00	15,89
12	1	2	22,00	17,00	40,00	42,00	99*	16,00	18,39*	36,53	34,00	99*
32	2	2	21,00	16,00	27,00	24,00	17,00	17,00	16,39	28,53	23,00	10,00
45	1	1	17,72*	16,00	43,00	40,00	23,00	20,00	18,00	45,00	34,00	25,00
18	1	1	25,00	18,00	53,00	46,00	20,00	20,00	16,00	50,00	41,00	16,00
20	1	2	15,00	20,00	34,00	26,00	19,00	14,00	17,00	29,00	26,00	14,00
34	2	2	19,00	18,27*	26,00	24,00	11,00	17,00	18,39*	28,00	21,00	11,00
64	1	2	19,00	19,00	33,00	22,00	15,09	21,00	18,00	27,00	24,00	14,00
11	1	2	17,00	21,00	33,00	28,00	9,00	17,00	21,00	26,00	24,00	10,00
49	1	1	23,00	21,00	38,00	33,00	12,00	19,00	21,39	35,53	34,00	10,00
48	1	2	18,00	18,00	32,00	31,00	24,00	16,00	18,00	40,00	33,00	28,00
19	1	1	21,00	20,00	44,00	38,00	12,00	19,00	19,00	47,00	35,00	16,00
27	1	1	22,00	19,00	45,00	37,00	18,00	21,00	20,00	43,00	41,00	19,00
10	1	2	17,00	18,00	33,00	27,72	9,00	17,00	20,39	25,53	24,00	10,00

41	2	1	20,00	20,00	24,00	25,00	15,00	19,00	22,00	28,00	26,00	14,00
60	2	2	19,00	21,00	30,00	26,41	8,00	19,00	21,00	37,00	28,00	16,00
35	2	2	21,00	19,00	39,00	37,00	13,00	21,00	18,00	41,00	38,00	16,00
59	2	1	19,00	21,00	33,00	25,00	8,00	19,00	20,00	37,00	28,00	16,00
54	2	2	25,00	20,00	58,00	51,00	15,00	18,00	22,00	42,00	41,00	12,00
63	2	2	22,00	17,35	60,00	51,72	16,00	19,00	17,00	54,00	49,00	17,00
38	1	1	25,00	18,00	49,00	53,00	22,00	21,00	13,00	56,00	60,00	28,00
30	1	2	25,00	19,00	40,00	33,00	15,00	22,00	19,00	33,00	34,00	15,00
46	2	2	22,03	17,00	41,00	35,26	23,00	18,08*	17,00	36,00	27,00	19,00
37	1	2	23,00	21,00	38,00	33,00	99*	20,00	20,00	29,00	34,22	99*
33	1	2	19,00	17,00	44,00	29,00	24,00	17,00	15,00	36,00	32,00	16,00
29	1	2	18,72*	18,00	31,00	27,00	19,15	17,00	18,00	36,53	28,00	12,89
31	2	2	21,00	16,00	27,00	23,72	17,00	17,00	17,00	30,00	23,00	10,00
56	1	1	22,00	18,00	99*	99*	17,00	21,00	19,00	99*	99*	20,00

Note: random: 1 = Music therapy group; 2 = Control group; sex: 1 = female; 2 = male; * = missing data
